# Up-regulation of miR-204 inhibits proliferation, invasion and apoptosis of gallbladder cancer cells by targeting Notch2

**DOI:** 10.18632/aging.202444

**Published:** 2021-01-13

**Authors:** Baohua Zhang, Haiyan Cui, Yinping Sun, Xinmei Wang, Qing Jia, Jing Li, Yingchun Yin, Xiaoyu Sun, Huirong Xu, Hongwei Li, Famei Xu, Jiansheng Rong

**Affiliations:** 1 Department of Pathology, Zibo Central Hospital, Zibo 255000, Shandong Province, China; 2 Department of Pathology, Zibo Fourth People’s Hospital, Zibo 255067, Shandong Province, China; 3 The Third Ward of Oncology Department, Zibo Central Hospital, Zibo 255000, Shandong Province, China; 4 The First Ward of Gastroenterology Department, Zibo Central Hospital, Zibo 255000, Shandong Province, China

**Keywords:** miR-204, gallbladder carcinoma, prognosis, Notch2

## Abstract

Gallbladder carcinoma (GC) is an extremely malignant gastrointestinal tumor, but relevant mechanisms are still under investigation. MicroRNA (miR) is differentially expressed in a variety of tumors. Here we explored miR-204 in patients with GC and related mechanisms. A GSE104165 chip was downloaded from the gene expression omnibus (GEO) for analysis. The qRT-PCR assay was used for quantifying miR-204 and Notch2 in the serum and tissues of the patients, and the patients were followed up for 3 years to analyze independent factors of prognosis. The CCK8, transwell, and flow cytometry assays were applied for analyzing proliferation, invasion, as well as apoptosis of cells, and the dual luciferase reporter (DLR) assay was adopted for determining the association of miR-204 with Notch2. MiR-204 was low in patients with GC, and it might serve as a diagnostic indicator for GC. In addition, patients with low e MiR-204 usually faced high rates of III+IV stage, distant metastasis, and low differentiation, and also showed a poor prognosis. DLR assay verified the targeted binding of miR-204 to Notch2 mRNA.

## INTRODUCTION

Gallbladder carcinoma (GC), a malignant tumor derived from gallbladder epithelium, is characterized by high malignancy, strong invasiveness, lymph node metastasis proneness, and poor prognosis. The incidence rate accounts for 80%-95% in biliary tract neoplasms [1, 2]. As the latest global tumor epidemiology survey shows, 2018 has seen about 220,000 new patients with GC and more than 150,000 patients dying from GC [3]. Compared with other gastrointestinal tumors, GC shows a relatively low morbidity and mortality, but its onset is relatively insidious, and it can spread widely in the early stage through direct infiltration, lymphatic metastasis and blood circulation [4, 5]. Most patients have entered the middle / late stage yet at diagnosis. The 5-year survival under 5% is mainly caused by the insufficient tumor screening and the lack of specific tumor markers for GC [6]. Therefore, we hope to find a new potential tumor marker for GC to improve this situation.

Non-coding RNA is RNA unable to encode proteins. As the sequencing level advances, people have found that many non-coding RNAs can affect biological processes via targeted regulation on downstream genes [7]. MicroRNA (miR), one short-chain non-coding RNA (17-25nt long), has a high-degree conservation [8]. Over the past few years, miR is reported to lower gene expression through binding to complementary sequences in the UTR of specific target mRNA to degrade or suppress target gene mRNA [9]. Many studies have pointed out that miR represses invasion and proliferation of tumor cells by regulating downstream target genes [10, 11]. MiR-204 belonging to the miR family is a tumor suppressor [12]. Liu et al [13] found that miR-204 regulated EMT by suppressing invasion / migration of gastric cancer through targeting snai1. Jiang et al [14] pointed out that miR-204 could participate in development of hepatocellular carcinoma by regulating SIRT1. However, miR-204 in patients with GC, and related mechanisms are still under investigation.

Notch 2 is a gene encoding one transmembrane protein that functions in multiple developmental processes and functions as a receptor for membrane bound ligands and interacts with adjacent cells. Alterations in Notch 2 are frequently linked to cancer and bladder urothelial carcinoma having the greatest prevalence of Notch 2 alterations. This study quantified miR-204 in GC cells, and transfected cells with over-expression and inhabiting sequence. As a result, miR-204 expression was lower in cases with GC. Mechanism study denoted that miR-204 regulates Notch 2 and suppresses EMT. As our data suggests, miR-204 is a promising diagnostic index for GC.

## RESULTS

### Clinical value of miR-204 in GC

**Figure 1:**
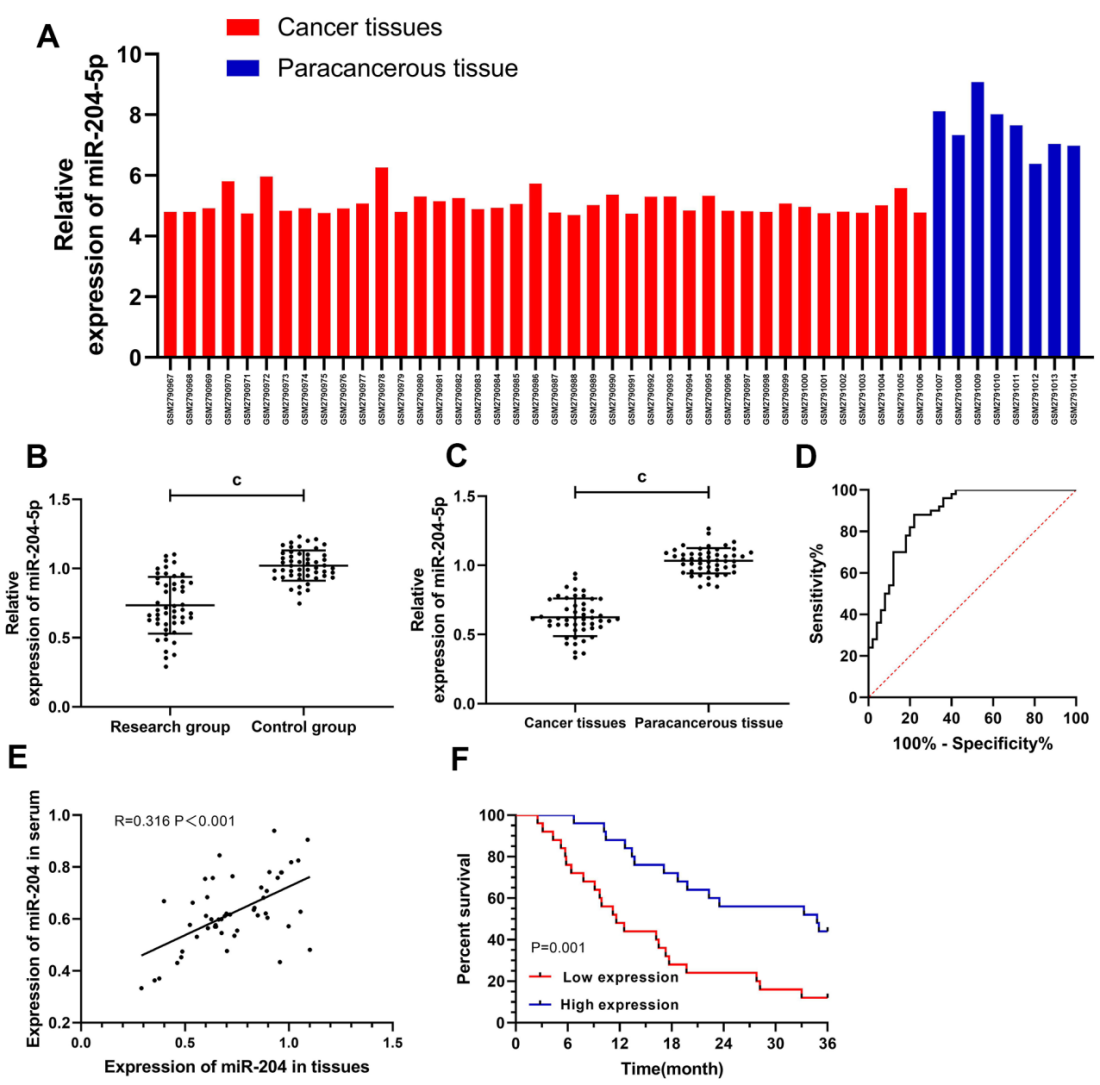
**Clinical value of miR-204 in gallbladder carcinoma.** (**A**) The expression of miR-204 in gallbladder carcinoma was decreased as detected by microarray; (**B**) The expression of miR-204 in patients serum from the study group was significantly decreased; (**C**) ROC curve of serum miR-204 in diagnosing gallbladder carcinoma, and the AUC was 0.884. When the cut-off value was 0.914, the optimal specificity, sensitivity and youden index was 78.00%, 88.00%, and 66.00%, respectively; (**D**) Expression of miR-204 in tissues was decreased; (**E**) Correlation analysis between miR-204 in serum and in tissues; (**F**) Patients with low expression of miR-204 showed a significantly lower 3-year survival rate than those with high expression (P=0.001). c indicates *p*<0.001.

Analysis of miR-204 in the patient tissues with GC based on the database (Table 1) uncovered that miR-204 decreased in the patients (Table 2 / Figure 1A). The clinical analysis revealed low miR-204 expression in both serum (Figure 1B) and tissues of the patients (Figure 1C), and the two were positively correlated (R=0.316, P<0.001) (Figure 1D). Based on ROC curve analysis, the AUC of miR-204 was 0.884, and patients with low miR-204 showed high rates of having III+IV stage, distant metastasis, and low differentiation (Figure 1E). Survival analysis uncovered notably lower 3-year survival among patients with low miR-204 (Figure 1F) and multivariate Cox regression analysis (Table 3) denoted distant metastasis and miR-204 underexpression as independent factors for patients’ prognosis.

**Table 1:** Data about microarrays.

**Data**	**GSE97332**
**Time**	
Submission date	Sep 22, 2017
Last update date	Apr 02, 2019
**Contact name**	Justo Lorenzo Bermejo
**Address**	
Organization name	Institute of Medical Biometry and Informatics, University of Heidelberg
Department	Statistical Genetics
Street address	Im Neuenheimer Feld, 130.3
City	Heidelberg
Country	Germany
**ZIP/Postal code**	310058
**Organism**	Homo sapiens
**Experiment type**	Non-coding RNA profiling by array
**Platforms**	Agilent-046064 Unrestricted_Human_miRNA_V19.0_Microarray (miRNA ID version)

**Table 2:** Relationship between miR-204 and pathological data about the patients.

**Factors**		**miR-204**		**χ^2^**	**P**
		**High expression (n=25)**	**Low expression (n=25)**		
Gender				1.754	0.185
	Male (n=38)	17 (68.00)	21 (84.00)		
	Female (n=12)	8 (32.00)	4 (16.00)		
Age (Y)				1.282	0.258
	<60 (n=26)	15 (60.00)	11 (44.00)		
	≥60 (n=24)	10 (40.00)	14 (56.00)		
Tumor size (cm)				2.013	0.156
	≥5 (n=27)	11 (44.00)	16 (64.00)		
	<5 (n=23)	14 (56.00)	9 (36.00)		
TNM staging			6.480	0.011
	I+II stage (n=25)	17 (68.00)	8 (32.00)
	III+IV stage (n=25)	8 (32.00)	17 (68.00)
Distant metastasis				9.934	0.002
	Yes (n=21)	16 (64.00)	5 (20.00)		
	No (n=29)	9 (36.00)	20 (80.00)		
Differentiation				5.333	0.021
	Low differentiation (n=20)	14 (56.00)	6 (24.00)		
	Moderate + high differentiation (n=30)	11 (44.00)	19 (76.00)		

**Table 3:** Cox regression analysis.

**Factors**	**Univariate Cox**			**Multivariate Cox**		
	**P**	**HR**	**95%CI**	**P**	**HR**	**95%CI**
Gender (male vs. female)	0.800	0.903	0.409-1.991			
Age (< 60 years vs. ≥ 60 years)	0.471	0.767	0.373-1.578			
Tumor size (≥ 5cm vs. < 5cm)	0.386	0.721	0.345-1.509			
TNM staging (I+II stage vs. III+IV stage)	0.011	0.391	0.189-0.807	0.029	0.454	0.224-0.922
Distant metastasis (yes vs. no)	0.267	1.521	0.726-3.187			
Differentiation (low vs. medium + high)	0.061	2.107	0.965-4.599	0.079	1.912	0.929-3.934
miR-204(High vs. Low)	0.110	1.950	0.859-4.427	0.034	2.215	1.06-4.63

### MiR-204 in GC cells, and its biological function

**Figure 2:**
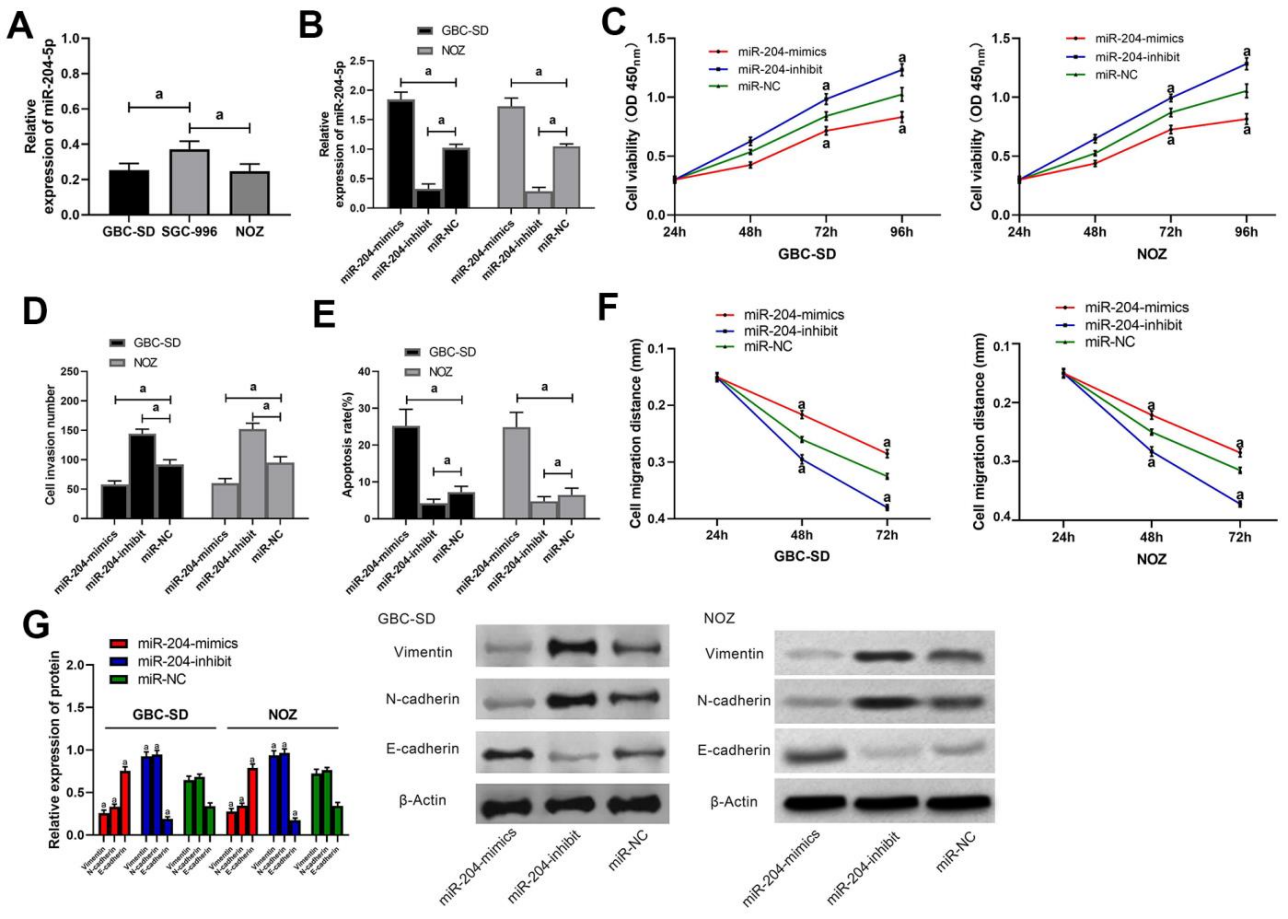
**Effects of miR-204 on biological functions of gallbladder carcinoma cells.** (**A**) Expression of miR-204 in GBC-SD, SGC-996, and NOZ cells; (**B**) Expression of miR-204 in GBC-SD and NOZ cells after transfection with miR-204-mimics, miR-204-inhibit and miR-NC, respectively; (**C**) Proliferation of GBC-SD and NOZ cells after miR transfection, as detected by CCK-8 assay; (**D**) Membrane penetration of GBC-SD and NOZ cells after miR transfection by transwell assay; (**E**) Apoptosis rate of GBC-SD and NOZ cells after transfection by flow cytometry; (**F**) Migration of GBC-SD and NOZ cells after transfection by cell scratch assay; (**G**) Expression of Vimentin, N-cadherin and E-cadherin in GBC-SD and NOZ cells by western blot. a indicates in comparison with the miR-NC group, *p*<0.05.

Comparison among GBC-SD, SGC-996, and NOZ cells revealed that GBC-SD and NOZ cells showed an obviously lower miR-204 (Figure 2A). Therefore, we selected the two cells for further experiments. Cells were transfected by miR-204 mimics/ miR-204 inhibit /miR-NC (Figure 2B). As a result, those transfected with miR-204-mimics presented notably lower proliferation, invasion as well as migration and notably higher apoptosis than those with miR-NC (Figure 2C–2F) and also showed lower Vimentin and N-cadherin and higher E-cadherin (Figure 2G). In addition, cells transfected with miR-204-inhibit presented notably faster proliferation, invasion (Supplementary Figure 1) as well as migration (Supplementary Figure 2A, 2B) and notably lower apoptosis than those with miR-NC (Figure 2C–2F) and also presented higher Vimentin and N-cadherin, and lower E-cadherin than the latter (Figure 2G).

### Relationship between miR-204 and Notch2

**Figure 3:**
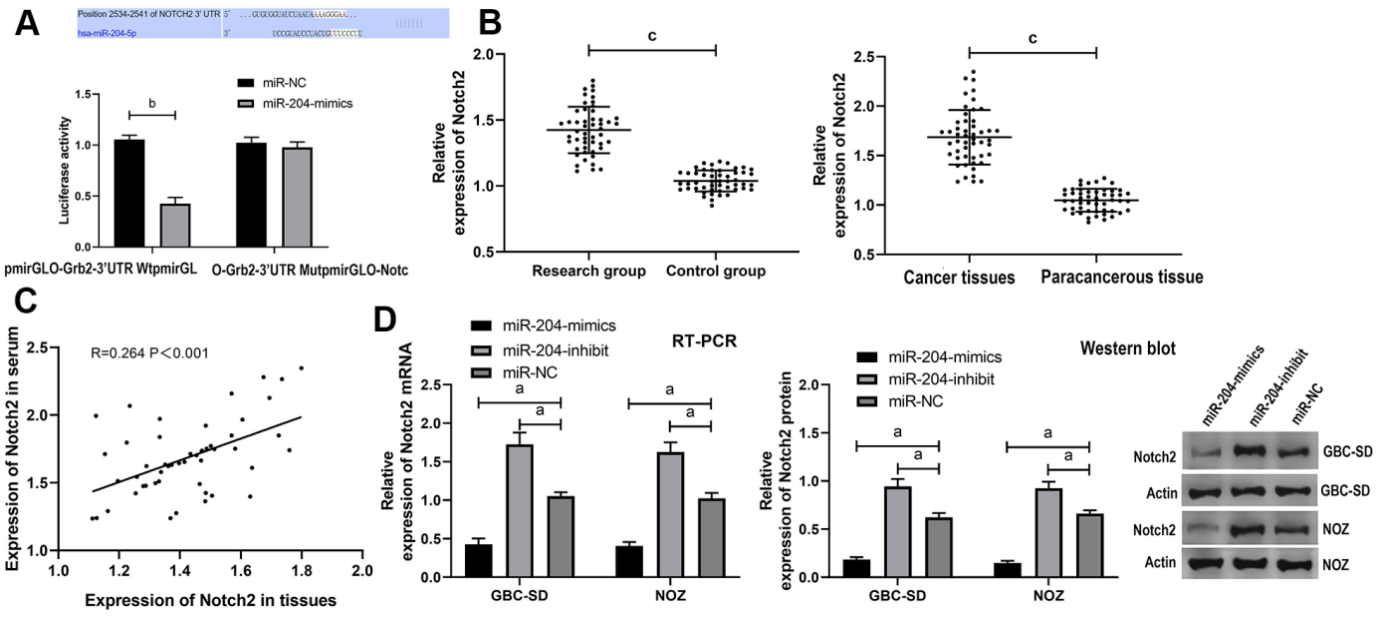
**Verification of targeting relationship between miR-204 and Notch2.** (**A**) DLR assay verified the targeting relationship between miR-204 and Notch2; (**B**) Expression of Notch2 protein in tissues and serum of the patients; (**C**) Correlation analysis between miR-204 in serum and in tissues of the patients; (**D**) Expression of Notch2 mRNA and protein in cells transfected with miR-204. a indicates *p*<0.05, b indicates *p*<0.01, and c indicates *p*<0.001.

Analysis through online predication websites (Targetscan7.2 / starBase 2.0 / miRBD / miRtarbase) revealed potential binding of miR-204 to Notch2. For further confirming the association between the two, we additionally conducted luciferase detection (Figure 3A), and acquired that over-expressing miR-204 caused notably decreased luciferase activity of pmirGLO-Notch2-3’UT Wt, while did not impact that of pmirGLO-Notch2-3’UTR Mut. Notch2 was up-regulated in both tissues and serum of the patients (Figure 3B), and the two were positively correlated (Figure 3C). According to qRT-PCR and western blot assays, cells processed with miR-204-mimics / miR-204-inhibitor were greatly different from those with miR-NC in Notch2 mRNA and protein (P<0.05) (Figure 3D).

### Notch2 in GC cells, and its effects on biological functions

**Figure 4:**
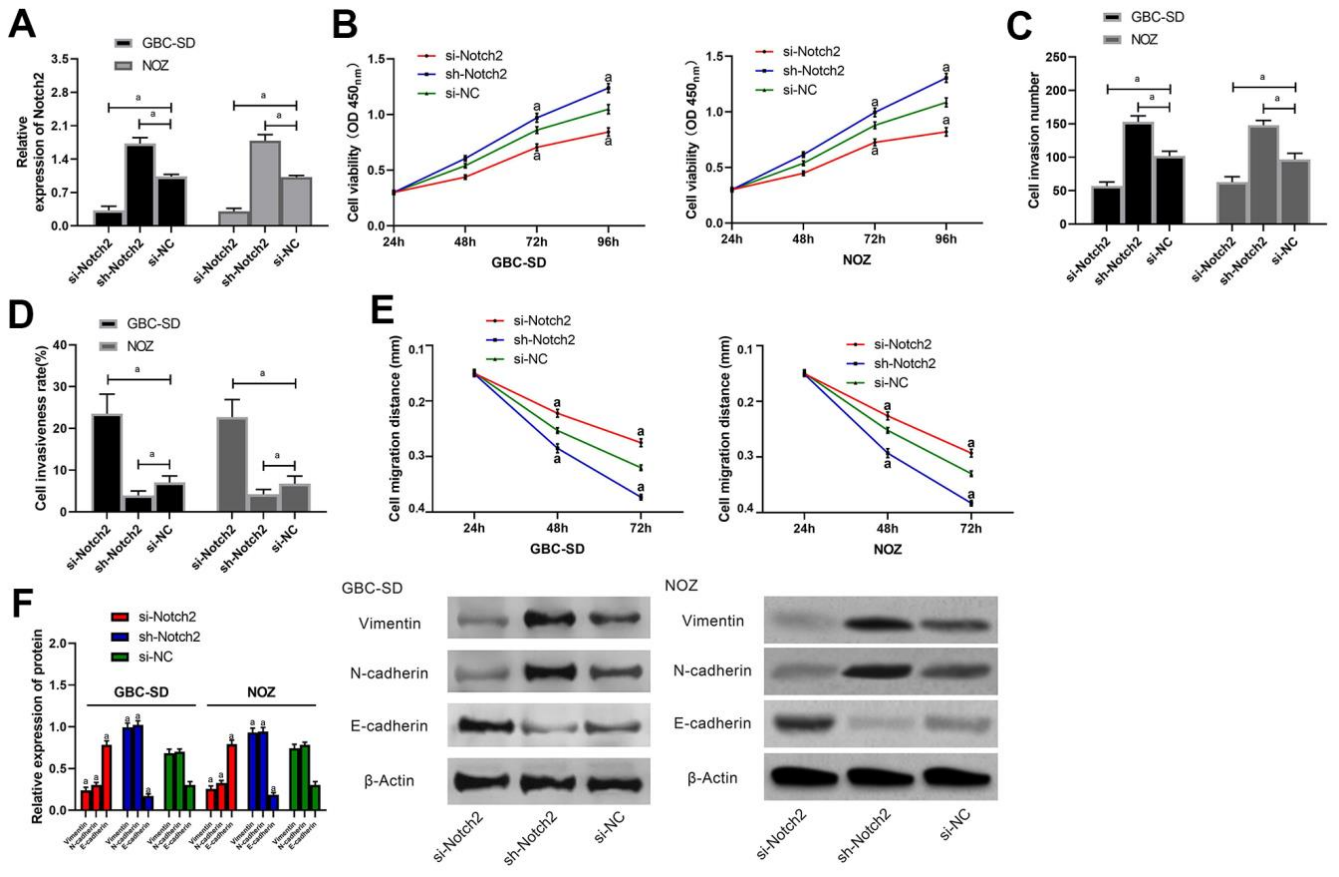
**Effects of Notch2 on biological functions of gallbladder carcinoma cells.** (**A**) Expression of Notch2 in GBC-SD and NOZ cells after miR transfection was detected by western blot; (**B**) Proliferation of GBC-SD and NOZ cells after transfection was measured by CCK-8 assay; (**C**) Membrane penetration of GBC-SD and NOZ cells after transfection was measured by transwell assay; (**D**) Apoptosis rates of GBC-SD and NOZ cells after transfection by flow cytometry; (**E**) Migration of GBC-SD and NOZ cells after transfection was analyzed by cell scratch assay; (**F**) Expression of Vimentin, N-cadherin and E-cadherin, in GBC-SD and NOZ cells after transfection by western blot. a indicates in comparison with the si-NC group, *p*< 0.05.

GBC-SD and NOZ cells were transfected with si-Notch 2, sh-Notch 2 or si-NC, and then the effects on Notch 2 were determined by qRT-PCR (Figure 4A). As a result, cells transfected with si-Notch2 (Notch2 knockdown) showed obviously lower proliferation, invasion as well as migration and greatly higher apoptosis than those with si-NC (control) (Figure 4B–4E) and also showed lower Vimentin and N-cadherin and higher E-cadherin than the latter (Figure 4F). In contrast, cells transfected with sh-Notch2 (Notch2 overexpression) presented greatly faster proliferation, invasion (Supplementary Figure 3) as well as migration (Supplementary Figure 4A, 4B) and greatly lower apoptosis than those with si-NC (Figure 4B–4E), and also presented higher Vimentin and N-cadherin and lower E-cadherin than the latter (Figure 4F).

### Rescue experiment

**Figure 5:**
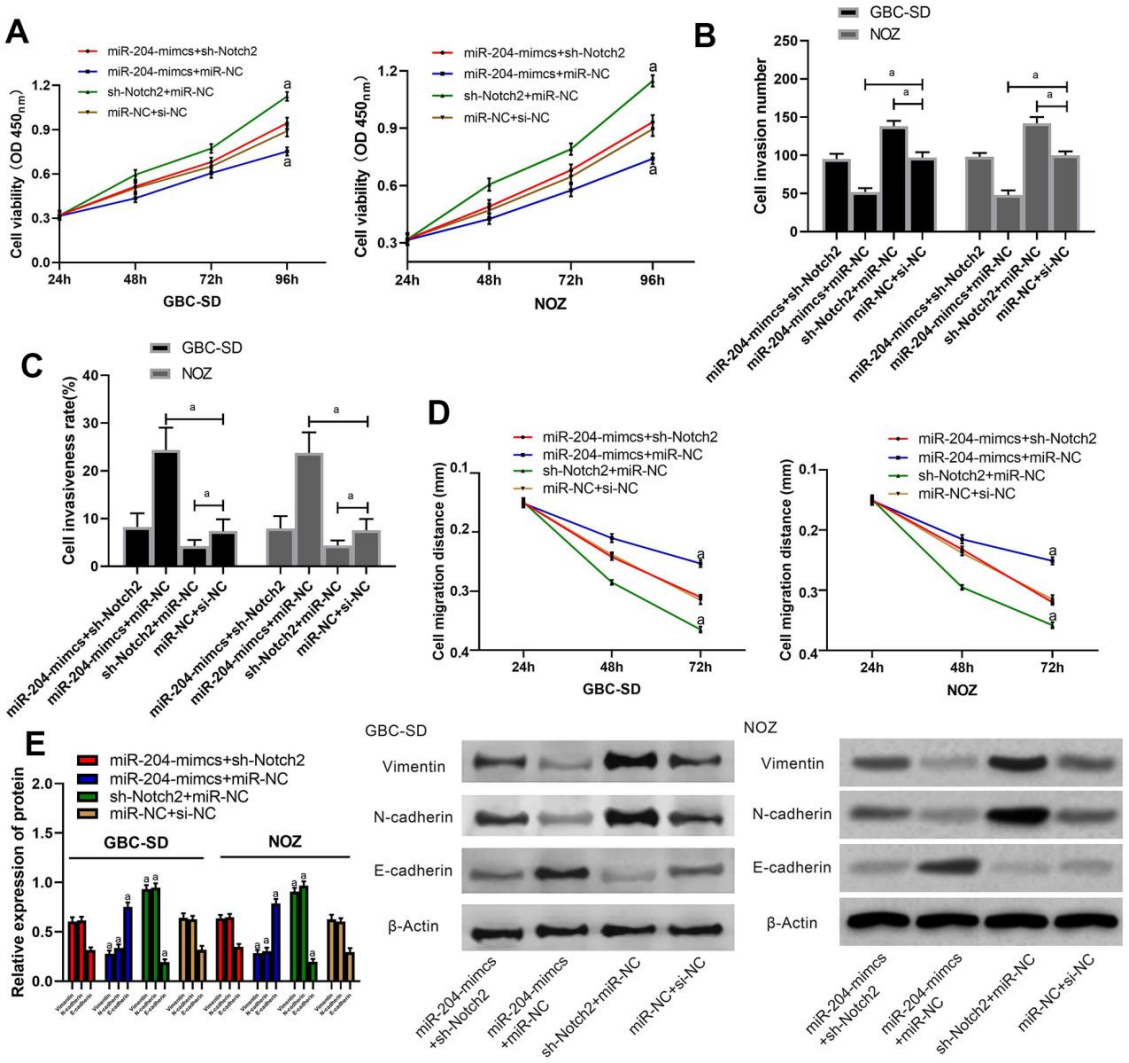
**Rescue experiment.** (**A**) Proliferation of cells transfected by various combination of miRs; (**B**) Membrane penetration of cells transfected by various combination of miRs; (**C**) Comparison of apoptosis rate; (**D**) Comparison of migration; (**E**) Protein levels of Vimentin, N-cadherin and E-cadherin as detected by western blot. a indicates in comparison with cells transfected with miR-NC+si-NC, p < 0.05.

We transfected miR-204-mimcs+sh-Notch2, miR-204-mimcs+miR-NC, sh-Notch2+miR-NC, and miR-NC+si-NC into NOZ and GBC-SD cells and determined biological functions of the cells (Figure 5A), invasion (Figure 5B, Supplementary Figure 5), migration (Figure 5D, Supplementary Figure 6A, 6B), apoptosis (Figure 5C) rate, and Vimentin, N-cadherin, and E-cadherin (Figure 5E) (all P > 0.05). Cells transfected with miR-204-mimcs+miR-NC presented notably slower proliferation, invasion as well as migration, and notably faster apoptosis than those with miR-204-mimcs+sh-Notch2 or miR-NC+si-NC, and showed lower N-cadherin and Vimentin and higher cadherin. In addition, cells transfected with sh-Notch2+miR-NC presented notably faster proliferation, invasion, as well as migration, and notably slower apoptosis than those with miR-204-mimcs+sh-Notch2 or miR-NC+si-NC, and also presented. higher N-cadherin and Vimentin and lower E-cadherin.

## DISCUSSION

GC is a severe malignancy. Based on statistical data, the 5-year survival of cases with GC is lower than 10%, and such a high mortality is one the problems needed to be solved urgently for clinicians [15]. We believe that such a high mortality is due to the following reasons: Firstly, GC is insidious at the onset, and it has no evident clinical features in the early phase, so it is prone to be neglected. Secondly, patients diagnosed with GC mostly have entered the middle or late stage yet, and are not eligible to surgery, and the prognosis of them after chemotherapy is poor. Finally, due to the limited GC screening, patients do not pay attention to the disease [16–18]. The above problems are mainly due to the lack of tumor markers specific to GC clinically, so it is particularly important to find such tumor markers with high specificity for GC.

The expression of CA199 in patients with one of many gastrointestinal tumors increases significantly. A previous study has found that CA199 has certain clinical value in diagnosis of GC, but its specificity is low [19]. Non-coding RNA is a RNA unable to encode proteins. MiR can regulate transcription of downstream target genes via the 3'-UTR end and plays a role in inhibiting and degrading target genes [20, 21] MiR-204, located on chromosome 9q21.12, is an important tumor suppressor gene in the miR family. According to earlier studies, miR-204 is underexpressed in patients with one of many gastrointestinal tumors [13, 22]. However, there are few studies on miR-204 in cases with GC. In our study, analysis of miR-204 based on the GEO showed that the expression was low in patients with GC, which indicated that miR-204 may become a potential marker for diagnosis of GC. To confirm it, we conducted clinical research, and found greatly decreased miR-204 in serum and tissues of the patients and a positively correlation between them. ROC curves revealed that the AUC of miR-204 was 0.884, suggesting that miR-204 had a good clinical value. Prognosis of GC has always been one of the difficult problems for clinicians. Previous studies have found that miR-34 is able to regulate the telomere length of tumor stem cells and is strongly bound up with poor prognosis of cases with GC [23]. We assigned the patients to high and low miR-204 expression groups for analyzing their 3-year survival, finding that patients with low miR-204 showed a notably lower 3-year survival. Additionally, Cox regression uncovered that miR-204 was an independent factor impacting patients’ prognosis, which suggested that lowly expressed miR-204 indicated poor prognosis of patients.

According to the above, we have preliminarily demonstrated the clinical value of miR-204 in GC, but the specific mechanism remains unclear. Notch signaling pathway widely exists in invertebrates and vertebrates, which can regulate cell proliferation, apoptosis and differentiation, and has an effect on the development of tissues and organs [24, 25]. EMT is a process in which epithelial cells lose their original polarity, thereby acquiring anti-apoptosis and strong migration and invasion abilities, and converting into interstitial cells [26]. A study found that Notch pathway acted as a pivotal part in EMT [27]. Notch2 is one of the important factors in Notch signaling pathway. Wang et al [28]. discovered that miR-146a-5p mediated EMT of esophageal squamous cell carcinoma through targeting Notch2. Our study quantified miR-204 and Notch2 in cells, and transfected cells with over-expression sequence and inhabiting sequence, finding that the proliferation / invasion / migration of cells with over-expressed miR-204 or inhibited Notch2 were suppressed and their apoptosis rate increased. Vimentin, N-cadherin as well as E-cadherin are all EMT markers [29]. In this study, we conducted WB assay and found that N-cadherin and Vimentin were down-regulated notably, and E-cadherin was up-regulated notably, which indicated that the progress of EMT could be inhibited by over-expressed miR-204 and inhibited Notch2 expression. In addition, we determined the targeted genes of miR-204, finding targeted sites in Notch2. To confirm it, we conducted DLR assay, finding the ability of miR-204 in regulating Notch2. When miR-204 was over-expressed, the expression of Notch2 was significantly decreased according to protein determination and mRNA determination. Finally, we conducted a rescue experiment, finding that cells co-transfected with miR-204-mimics and sh-Notch2 were similar to those with miR-NC+si-NC in proliferation, invasion, migration as well as apoptosis. The results denoted that miR-204 could regulate Notch2 and curb the progress of EMT.

Based on this study, our data indicated that miR-204 can suppress EMT by regulating the expression of Notch2. However, this study still has certain limitations. First, we have not conducted nude mouse tumorigenesis test, and whether over-expression of miR-204 impacts tumor size in nude mice is unclear. Secondly, this study has not determined Notch signaling pathway, and have only determined the expression of Notch2, so the specific approach needs more deeply verification. Finally, due to the short time of this study, the time of follow-up only spans 3 years, and whether miR-204 has a long-term prognostic value in GC requires further exploration. Thus, we hope to carry out more basic experiments and follow up patients for a long time in the future to address our study deficiencies.

## CONCLUSIONS

To sum up, the prognosis of cases with GC and decreased miR-204 was poor, and miR-204 inhibits EMT by regulating Notch2.

## MATERIALS AND METHODS

### GEO data downloading

We selected GSE104165 microarrays through primary screening by searching miR-related microarrays for GC in https://www.ncbi.nlm.nih.gov/gds/. Then we analyzed the first 250 differential miRs in microarrays using GEO2R provided by the platform, and selected miR-204 for this study according to the analysis and references. Data of microarrays are shown in Table 1.

### Collection of clinical samples

Totally 50 patients with GC (38 males and 12 females, 58.9±5.0 years on average) admitted to Zibo Central Hospital between March 2014 and September 2015 were assigned to a study group, and 50 healthy individuals (40 males and 10 females, 59.2±4.5 years on average) were assigned to a normal group meantime. There were no statistical differences between them in age (P>0.05). Inclusion criteria: patients who provided written informed consent after comprehending the study, patients diagnosed as GC via pathology and imaging, and those conforming to the TNM staging criteria for GC (8^th^ edition) [30]. Exclusion criteria: Patients with other comorbid tumors, patients who had been targetedly treated against tumors before this study, patients with other comorbid congenital diseases or with estimated survival under 1 month, and patients unwilling to receive the follow-up. This study was carried out with approval from the Ethics Committee of our hospital and in accord with the Declaration of Helsinki.

### Collection of specimens

Tumor and tumor-adjacent tissues were acquired from patients during operation. For those who did not meet surgery conditions, the tissues were sampled during pathologic biopsy with their consent after communication. Before collecting tissues, peripheral blood (5 mL) was sampled from each patient, let stand for 30 min, and then subjected to 10-min centrifugation (3000 rpm) for collecting serum for subsequent analysis. Blood from healthy persons was collected and stored similarly as the patients to rule out possible artifacts due to sampling or storage.

### Cell sources

GBC-SD and SGC-996 cells were offered by Shanghai Institute of Nutrition and Health, Chinese Academy of Sciences and NOZ cells by BeNa Culture Collection, Beijing, CN.

### Main reagents and instruments

Primary antibodies of Notch2 (ab8926), Vimentin (ab137321), E-cadherin (ab1416), N-cadherin (ab18203), β-actin (ab8227), and HRP-labeled goat anti-mouse IgG secondary antibody (ab10183) were offered by Abcam, USA. RIPA kit (89900), BCA protein kit (23250), ECL kit (32209), trypsin (90059), and Lipofectamine™ 2000 Transfection Reagent (11668019) were offered by Thermo Scientific, USA. TransScript II Green Two-Step qRT-PCR SuperMix (AQ301-01) and TransScript Green miRNA Two-Step qRT-PCR SuperMix (AQ202-01) were from TransGen Biotech (Beijing, CN). CCK8 was from Beyotime Biotechnology, (Shanghai, CN). Transwell kit (1142802), DMEM (10566024), PBS (10010023), FBS (26400044), and penicillin-streptomycin (15070063) were offered by Gibco, USA. Annexin V/PI apoptosis determination kit was offered by Shanghai Ye Sen Biological Technology. DLR gene detection kit was from Solarbio Company (Beijing, CN). RT-PCR was performed in an ABI 7500 thermal cycler. BD FACS Canto II was used for flow cytometry. Shanghai GenePharm took charge of the design and synthesis of all primers.

### Cell incubation

GBC-SD, SGC-996, as well as NOZ cells were incubated in DMEM containing10% FBS, 1X penicillin-streptomycin, in a humidified incubator at 37° C under 5% CO_2_. The cells were transfected with Lipofectamine™ 2000 kit to construct miR-204-mimics (over-expression sequence), miR-204-inhibitor, miR-NC, sh-Notch2, si-Notch2, and si-NC strictly under the kit guidance.

### qRT-PCR

We used a TRIzol kit for extracting the total RNA of the collected samples, and determined its concentration, purity as well as integrity through an ultraviolet spectrophotometer and agarose gel electrophoresis, and then conducted reverse transcription strictly under the kit guidance. Three duplicate wells were set for every specimen, and each specimen was determined 3 times, with U6 and GAPD as internal references for miR and mRNA. Data of this experiment were analyzed using 2^-ΔΔct^ [31].

### Western blot

We collected incubated cells of every group, extracted their total protein via RIPA lysis, and determined the protein concentration via the BCA means. The total protein was isolated via 12% SDS-PAGE, and placed on one PVDF membrane. Primary antibodies of Caspase3, Bcl-2, Notch2, Bax, E-cadherin and β-catenin were used at 1: 1000 dilution. Horseradish peroxidase labeled goat anti-rabbit secondary antibody was used at 1: 5000 dilution. ECL chemiluminescent kit was used for detection of target bands. The protein band was scanned, and its gray value was calculated by Quantity One. The amount of protein was normalized to β-Actin protein band.

### Cell proliferation determination

Cells transfected for 24 hours were placed on a 96-well plate at 4x10^6^ cell/well, and every well was put with 10 μL CCK-8 solution and 90 μL basic DMEM at 0, 24, 48, and 72 h after placement, and the plate was then subjected to culture (37° C, 2 h). The optical density of every well at 450 nm was measured via one microplate reader.

### Cell scratch assay

The transfected cells were placed on one 6-well plate at 2x10^5^ cells / well. After 24 h, a line was drawn in the plate along the diameter of it, and PBS was used to gently wash the floating cells. Five randomly selected fields of view under a 20-fold microscope were photographed for each well. The plate was cultured using a serum-free medium, and then photographed for each well in the same way at 24 h and 48 h, respectively.

### Cell invasion and detection

Cells transfected for 24 h were placed on one 6-well plate at 5x10^4^ cells/well. The cells (cleaned via PBS) and DMEM (200 μL) were placed in the upper compartment, and 500 μL DMEM with 20% FBS was placed in the lower one. The plate was subjected to culture (48 h, 37° C), and the substrate and cells failing to penetrate the membrane were wiped off. The plate was cleaned 3 times, followed by 10-min immobilization via paraformaldehyde, and 3 times of washing via double distilled water. The invasive cells in the plate were dyed by 0.5% crystal violet and analyzed using one microscope.

### Apoptosis determination

Cells were labeled with AnnexinV-FITC and PI successively, followed by 5-min incubation at room temperature with dark surroundings and detection via the FC500MCL flow cytometer. The cells were determined 3 times and the results were averaged.

### Prediction of target genes

MiRBD / Targetscan 7.2 / starBase 2.0 / miRtarbase were used for predicting the downstream target genes of miR-204. PmirGLO-Notch2-3’UTR wild type (Wt), miR-204-mimics, pmirGLO-Notch2-3’UTR mutant type (Mut), or miR-NC were transferred to HEK293T cells with a Lipofectamine™ 2000 kit. The luciferase activity was tested with the DLR gene assay kit.

### Statistical analyses

This study adopted SPSS20.0 for data analyses and GraphPad 7 for graphing. The K-S test was adopted for evaluation of the distribution of measurement data, and data in normal distribution were represented by the Mean ± SD. Inter-group comparison was performed via the independent-samples T test. Data not in normal distribution were presented via the quartile [Mean (P_25_-P_75_)], studied via the nonparametric test, and presented by Z. Multi-group comparison was conducted via the one-way ANOVA, and presented via F. The LSD-t test was adopted for Post hoc pairwise comparison. Data in multiple time points was compared via the variance of repeated measures, and presented by F. Bonferroni was adopted for Post test. Additionally, ROC curves of miR-204 and Notch2 in diagnostic significance for GC were drawn, and the correlation of expression of miR-204 in serum with that in tissues was analyzed via the Pearson correlation coefficient. Figures about the 5-year survival of cases were drawn using Kaplan Mayer survival curves, and studied via the Log-rank test. Multivariate Cox regression was adopted for prognosis analysis of patients. P < 0.05 indicates a notable difference.

## Supplementary Material

Supplementary Figures
