# Supplementary material for: Up-regulation of miR-204 inhibits proliferation, invasion and apoptosis of gallbladder cancer cells by targeting Notch2
Source: Aging (Albany NY). 2021 Jan 13;13(2):2941–58. doi: 10.18632/aging.202444 (PMC7880336; doi:10.18632/aging.202444)
Supplement: Supplementary Figures [file aging-13-202444-s001.pdf]

SUPPLEMENTARY FIGURES

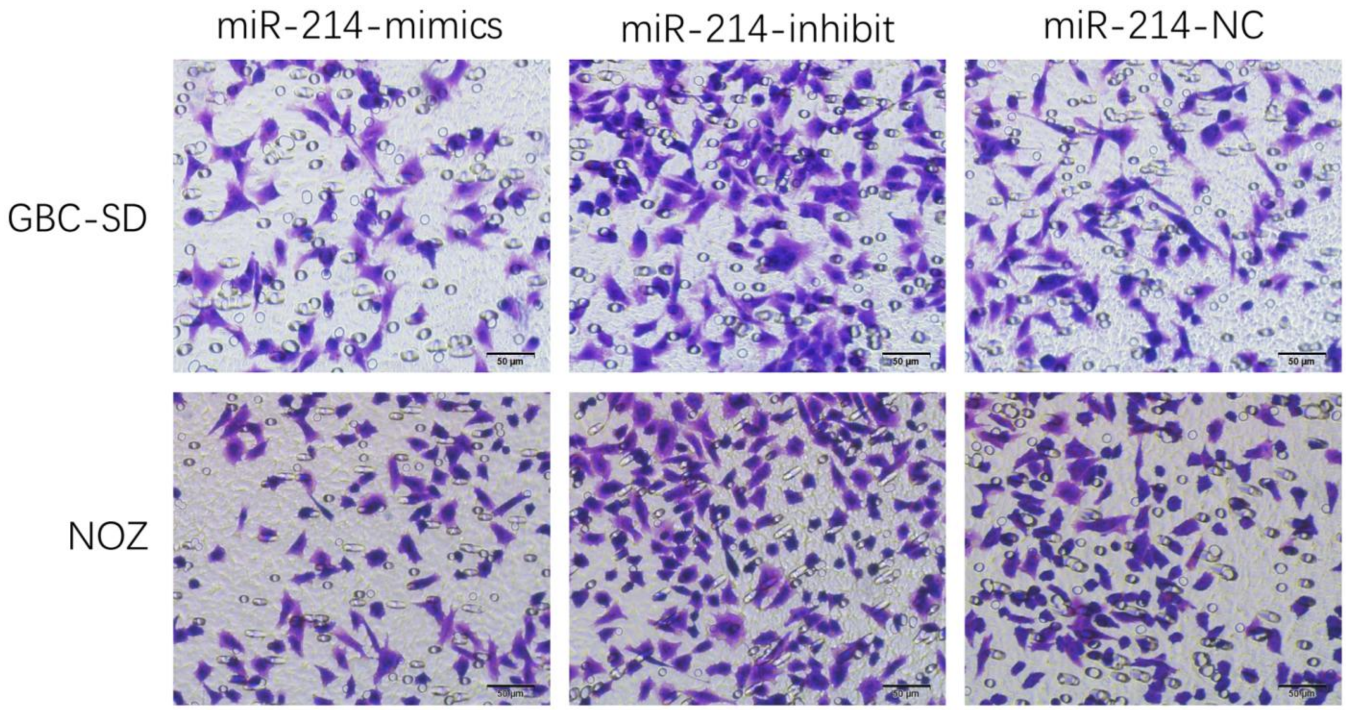

Supplementary Figure 1. Cell invasion assay in transwell chambers by using GBC-SD or NOZ cell lines transfected with miR-214-mimics, miR-214-inhibit and miR-214-NC control. Cells were stained with crystal violet and photographed (magnification, ×100). Black bars indicate 50 m. Compared with the control cells, cells with miR-214 knockdown (miR-214-inhibit) showed significantly enhanced migration and invasion capacity, while overexpression of miR-214 (miR-214-mimics) inhibited cell invasion.

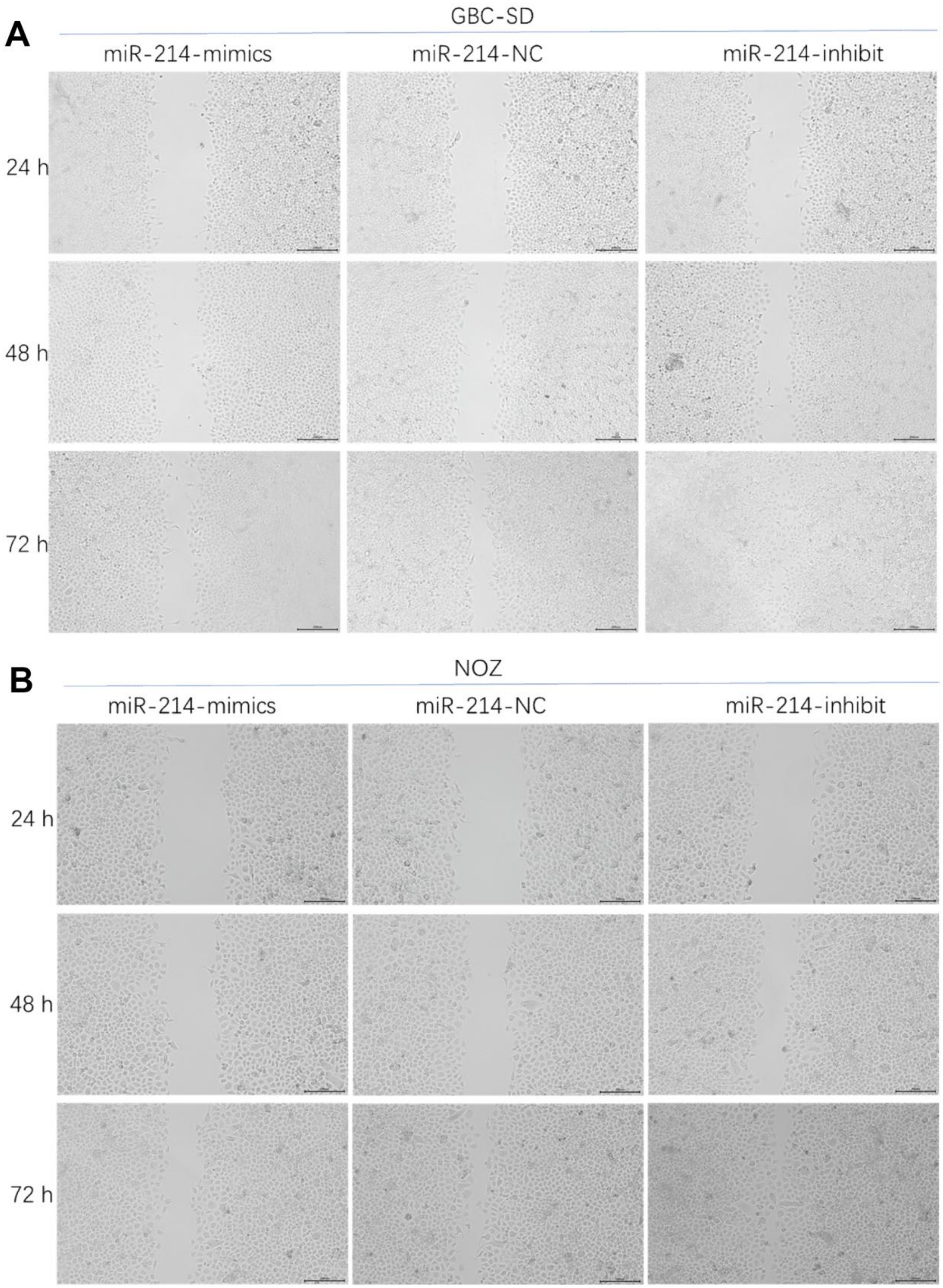

Supplementary Figure 2. Cell migration assay using GBS-SD (**A**) and NOZ (**B**) cell lines transfected with miR-214-mimics, miR-14-inhibit and miR-214-NC control. Scratch assay was made on confluent cells by drawing a line across the bottom of the dish. The figure shows micrographs of the extent of closure obtained after 24, 48 and 72 hours under different transfection conditions. Phase-contrast microscopy pictures were taken of the wounded area. Black bars indicated 200 μm.

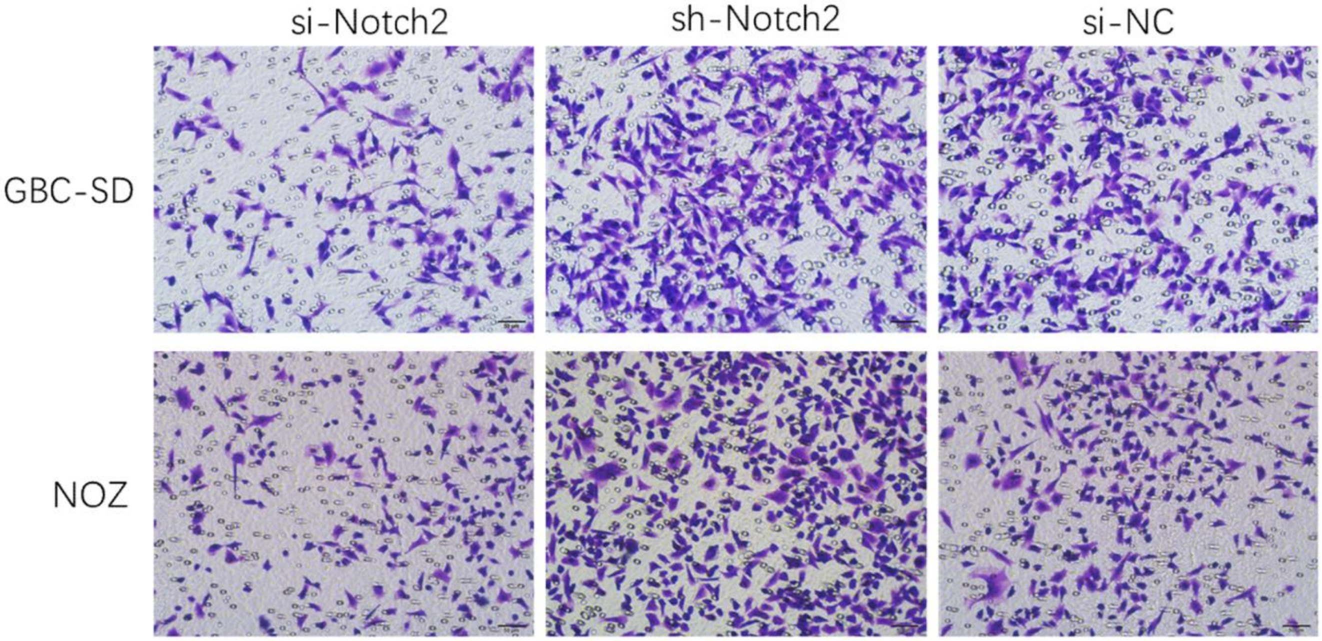

Supplementary Figure 3. Cell invasion assay in transwell chambers by using GBC-SD or NOZ cell lines transfected with si-Notch2, sh-Notch2 and si-NC control. Cells were stained with crystal violet and photographed (magnification, ×100). Black bars indicate 50 m. Compared with the control cells, cells with Notch2 overexpression (sh-Notch2) showed significantly enhanced migration and invasion capacity, while knockdown of Notch2 (si-Notch2) inhibited cell invasion.

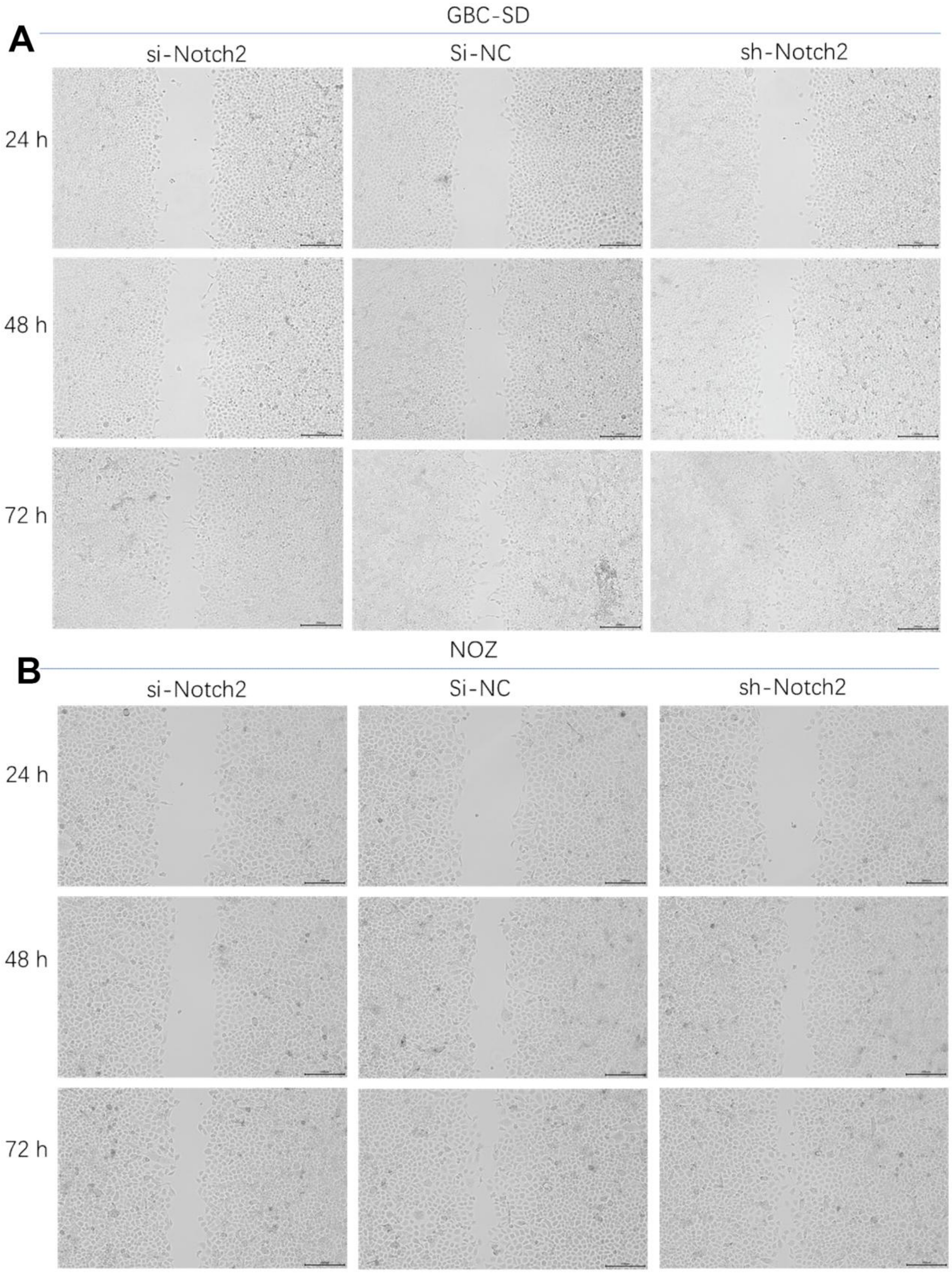

Supplementary Figure 4. Cell migration assay using GBS-SD (**A**) and NOZ (**B**) cell lines transfected with si-Notch2, sh-Notch2 and si-NC control. Scratch assay was made on confluent cells by drawing a line across the bottom of the dish. The figure shows micrographs of the extent of closure obtained after 24, 48 and 72 hours under different transfection conditions. Phase-contrast microscopy pictures were taken of the wounded area. Black bars indicated 200 µm.

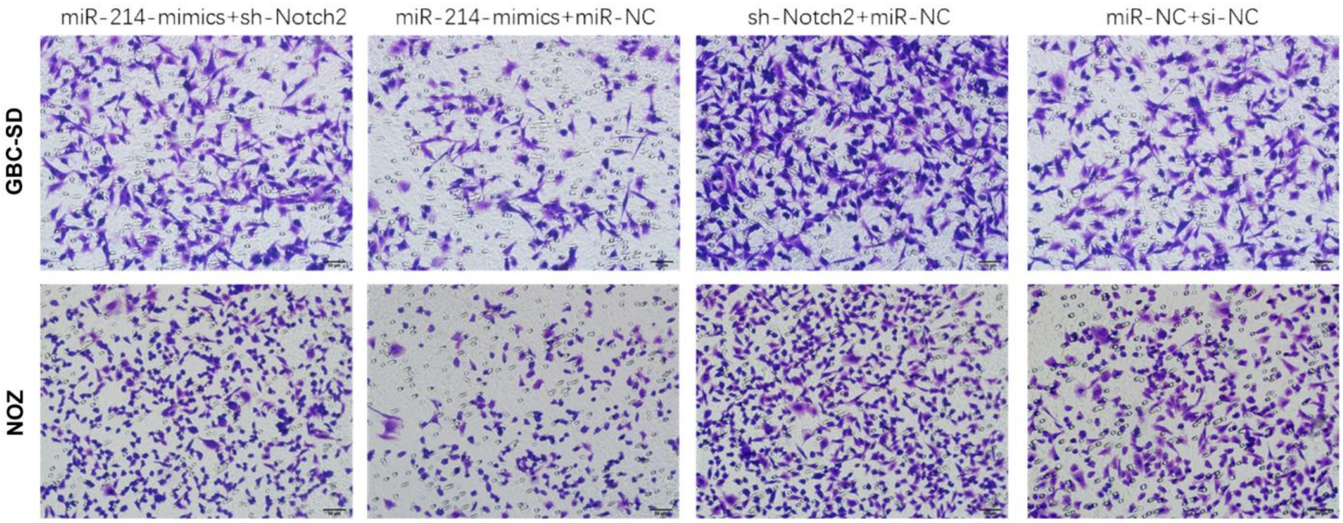

Supplementary Figure 5. Cell invasion assay in transwell chambers by using GBC-SD or NOZ cell lines transfected with miR-214-mimics+sh-Notch2, miR-214-mimics+miR-NC, sh-Notch2+miR-NC and miR-NC+si-NC. Cells were stained with crystal violet and photographed (magnification, ×100). Black bars indicate 50 m.

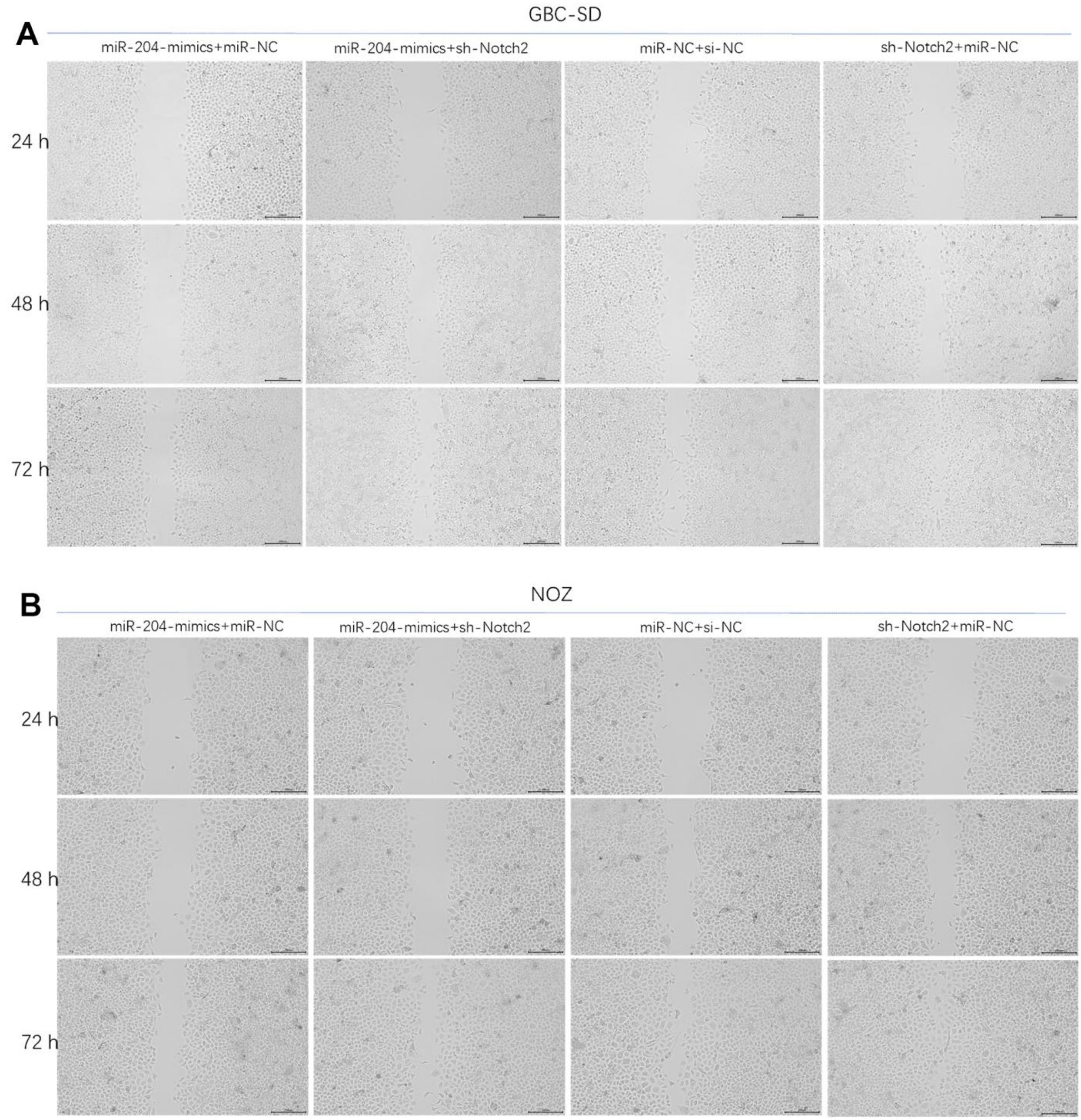

Supplementary Figure 6. Cell migration assay using GBS-SD (**A**) and NOZ (**B**) cell lines transfected with miR-214-mimics+sh-Notch2, miR-214-mimics+miR-NC, sh-Notch2+miR-NC and miR-NC+si-NC. Scratch assay was made on confluent cells by drawing a line across the bottom of the dish. The figure shows micrographs of the extent of closure obtained after 24, 48 and 72 hours under different transfection conditions. Phase-contrast microscopy pictures were taken of the wounded area. Black bars indicated 200 µm.
